# Supplementary material for: Identification of photoperiod-induced specific miRNAs in the adrenal glands of Sunite sheep (Ovis aries)
Source: Front Vet Sci. 2022 Jul 22;9:888207. doi: 10.3389/fvets.2022.888207 (PMC9354845; doi:10.3389/fvets.2022.888207)
Supplement: Supplementary file 1 [file Data_Sheet_1.ZIP › Supplementary file/miRDeep2 file.zip!/miRDeep2 file/Know_Structure/LP42A2/oar-mir-136.pdf]

miRBase precursor : oar-mir-136
Total read count : 9645
oar-miR-136 read count : 2964
remaining reads : 6681

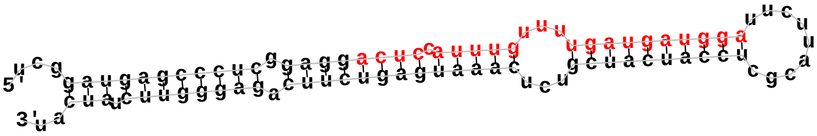

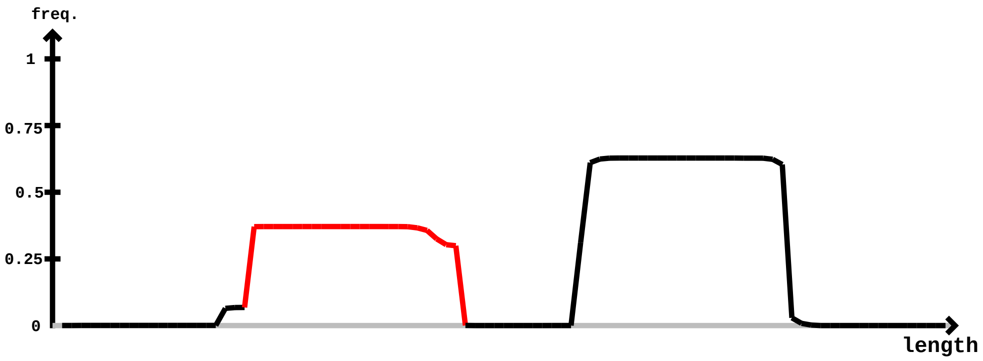


```
                   oar-miR-136
5'- ucggaugagcccucggaggacuccauuuguuuugaugauggauucuuacgcuccaucaucgucucaaaugagucuucagaggguucuaucau  -3'   exp
    ...(((((((((((.((((((((.(((((...(((((((((...........)))))))))...))))))))))))).)))))))).)))..    reads   mm   sample
    ucggaugagcccucggagg.........................................................................    2       0    seq
    .cggaugagcccucggagg.........................................................................    3       0    seq
    ................aggacuccauuuguuuC...........................................................    1       1    seq
    ................aggacuccauuuguuuuga.........................................................    5       0    seq
    ................aggacuccauuuguuuugau........................................................    42      0    seq
    ................aggacuccauuuguuuugaug.......................................................    90      0    seq
    ................aggacuccauuuguuuugauga......................................................    295     0    seq
    ................aggacuccauuuguuuugaugau.....................................................    183     0    seq
    ................aggacuccauuuguuuugaugaug....................................................    1       0    seq
    .................ggacuccauuuguuuugaug.......................................................    2       0    seq
    .................ggacuccauuuguuuugauga......................................................    10      0    seq
    .................ggacuccauuuguuuugaugau.....................................................    18      0    seq
    ..................gacuccauuuguuuugaugaugg...................................................    3       0    seq
    ...................acuccauuuguuuugaugau.....................................................    10      0    seq
    ...................Ccuccauuuguuuugaugaug....................................................    1       1    seq
    ...................acuccauuuguuuugaugaug....................................................    34      0    seq
    ...................acuccauuuguuuugaugaugg...................................................    2877    0    seq
    ...................acuccauuuguuuugaugaugga..................................................    5       0    seq
    ....................cuccauuuguuuugaugaug....................................................    2       0    seq
    ....................cuccauuuguuuugaugaugg...................................................    1       0    seq
    ........................auuuguuuugaugaugg...................................................    1       0    seq
    ..................................................cuccaucaucgucucaaaugagucu.................    1       0    seq
    .....................................................caucaucgucucaaaug......................    4       0    seq
    .....................................................caucaucgucucaaaugag....................    1       0    seq
    .....................................................caucaucgucucaaaugagu...................    32      0    seq
    .....................................................caucaucgucucaaaugaguc..................    138     0    seq
    .....................................................caucaucgucucaaaugagucu.................    2789    0    seq
    .....................................................caucaucgucucaaaugagucuu................    37      0    seq
    .....................................................caucaucgucucaaaugagucuuc...............    1       0    seq
    ......................................................aucaucgucucaaaugagu...................    8       0    seq
    ......................................................aucaucgucucaaaugagC...................    1       1    seq
    ......................................................aucaucgucucaaaugaguc..................    49      0    seq
    ......................................................aGcaucgucucaaaugagucu.................    1       1    seq
    ......................................................aucaucgucucaaaugagucu.................    2710    0    seq
    ......................................................aucaucgucucaaaugagucuu................    112     0    seq
```

oar-miR-136

```
ucggaugagcccucggaggacuccauuuguuuugaugauggauucuuacgcuccaucaucgucucaaaugagucuucagaggguucuaucau
......................................................aucaucgucucaaaugagucuuc...............    8    0    seq
.......................................................ucauUgucucaaaugagucu.................    1    1    seq
.......................................................ucaucgucucaaaugagucu.................    41   0    seq
.......................................................ucaucgucucaaaugagucuu................    49   0    seq
.......................................................ucaucgucucaaaugagucuuc...............    32   0    seq
.......................................................ucaucgucucaaaugagucuuca..............    7    0    seq
........................................................caucgucucaaaugagucuu................    1    0    seq
........................................................caucgucucaaaugagucuuc...............    20   0    seq
........................................................caucgucucaaaugagucuuca..............    12   0    seq
.........................................................aucgucucaaaugagucu.................    3    0    seq
.........................................................aucgucucaaaugagucuu................    1    0    seq
```
